# Detection of extraprostatic extension of prostate cancer by transfer learning AI models versus radiologists on biparametric and multiparametric MRI

**DOI:** 10.21203/rs.3.rs-11001056/v1

**Published:** 2026-10-05

**Authors:** Angela Tong, Alexander Daniels, Luke Ginocchio, Paul Smereka, Tarun Dutt, Lavanya Umapathy, Madhur Nayan, Hersh Chandarana, Patricia Johnson

**Affiliations:** NYU Grossman School of Medicine; University of Washington; NYU Grossman School of Medicine; NYU Grossman School of Medicine; NYU Grossman School of Medicine; NYU Grossman School of Medicine; NYU Grossman School of Medicine; NYU Grossman School of Medicine; NYU Grossman School of Medicine

**Keywords:** Prostate cancer, MRI, Deep learning, Extraprostatic Extension, Biparametric MRI, Multiparametric MRI

## Abstract

**Objectives:**

To develop and test deep-learning models which detect extraprostatic extension (EPE) on biparametric (bpMRI) and multiparametric MRI (mpMRI) of the prostate in comparison to radiologists.

**Methods:**

Consecutive patients at a large healthcare enterprise who underwent prostate MRI (2015 to 2023) with subsequent radical prostatectomy within 1 year were included. The dataset was divided into training/validation/test sets. Transfer learning models, composed of two multi-branch 3D convolutional neural networks based on a 3D ResNet-50 backbone, were trained on bpMRI (AI_bp_) with an input of axial T2-weighted imaging (T2WI) and diffusion-weighted imaging (DWI) and on mpMRI (AI_mp_) with an input of axial T2WI, axial DWI, and axial post-contrast imaging. No prostate gland or tumor segmentations were performed. A logistic regression model with prostate specific antigen density was also trained with the AI_mp_ model (AI_mpPSAD_). Three fellowship-trained abdominal radiologists evaluated the test set for EPE on a 1 to 5 scoring system based on capsular appearance, on bpMRI and mpMRI for each study. Areas under the receiver operating characteristic curves (AUC) were obtained and compared with Delong test. Diagnostic statistics were also obtained.

**Results:**

1,232 prostate MRIs were included (1003/113/116 in the training/validation/test sets). AUC for models were as follows: AI_bp_ (0.71), AI_mp_ (0.72), and AI_mpPSAD_ (0.73). While holding sensitivity constant at the Youden index for AI_bp_ (0.77), specificity and accuracy of the AI_bp_, AI_mp_, and AI_mpPSAD_ were 0.64, 0.69, 0.71 and 0.71, 0.73, 0.74, respectively. The ranges of AUC for readers were: bpMRI (0.66–0.71) and mpMRI (0.69–0.74). The ranges of accuracy of the readers on bpMRI was 0.60–0.68 and on mpMRI was 0.65–0.72.

**Conclusion:**

A segmentation-free transfer learning model performs comparably to fellowship-trained radiologists on the detection of EPE on both bpMRI and mpMRI. There was a trend of increasing accuracy with the addition of post contrast sequences for all models and readers.

## Introduction

Prostate cancer is the most common cancer among men in the United States, and accurate staging is critical for guiding management [1]. A key feature of advanced local disease is extraprostatic extension (EPE), defined as tumor spread beyond the prostate’s fibromuscular pseudocapsule [2]. The presence of EPE is an established risk factor for biochemical recurrence and metastasis after radical prostatectomy [3, 4]. Clinically, identifying EPE before or during treatment planning is crucial because it often warrants changes in management, including wider resection margins or foregoing nerve-sparing techniques. Adjuvant therapies like radiation or androgen deprivation may be recommended as well [5-7]. Further, it is an important prognosticator of prostate cancer. Men with EPE after radical prostatectomy had a more than fivefold increased risk of death from prostate cancer compared to those without EPE, even after adjusting for other factors such as Gleason score and PSA level [8]. Reliable pretreatment detection of EPE in prostate cancer is of high clinical significance.

Both multiparametric magnetic resonance imaging (mpMRI), with T2 weighted imaging (T2WI), diffusion weighted imaging (DWI), and post-contrast imaging especially dynamic contrast enhanced imaging (DCE), and biparametric MRI (bpMRI), including T2WI and DWI, have been shown to be effective in identifying and risk stratifying prostate lesions [9, 10]. They are also the modality of choice for local staging of prostate cancer including EPE, however, mpMRI only demonstrates moderate sensitivity and moderate-to-high specificity for detecting EPE, with most meta-analyses and large studies reporting sensitivity in the range of 32–58% and specificity in the range of 73–89% [11-13]. A recent trial which determined that bpMRI was non-inferior to mpMRI in detecting and risk stratifying prostate lesions also noted that EPE evaluation on mpMRI and bpMRI had 86.7% agreement but was unable to investigate true detection of EPE given the lack of prostatectomies in the trial [14].

Grading systems have been proposed to standardize MRI reads, taking into account features such as the length of capsular contact, capsular bulge/irregularity, and neurovascular bundle asymmetry to determine the likelihood of EPE [15, 16]. Even with grading systems, there is room for improvement in the detection of EPE.

Machine learning techniques may be leveraged to meet this need. Recent advances in machine learning show promise for improving EPE detection on prostate MRI when compared to radiologists especially when using deep learning models and radiomics [17-19]. The reported models require tumor and gland segmentations, which is a time-consuming process and demonstrate inter-reader variability especially with segmentation of lesions [20]. Given the clinical importance of detecting EPE and the limitations of segmentations, there is a clear need for more creative and improved diagnostic strategies. In this study, we build and test weakly supervised sequential transfer learning models to detect EPE on bpMRI and mpMRI compared with radiologists without the rate limiting step of prostate gland or tumor segmentation. We also assess the added value of DCE in detecting EPE to both readers and deep learning algorithms.

## Materials and Methods

This study is an institutional review board approved, HIPAA compliant study. Consent was waived as it was a retrospective study.

### Patients

The picture archive and communication system (PACS) was searched from December 1, 2015 to December 28, 2023 for adult patients who had a prostate MRI with the following inclusion criteria: 1. Clinical suspicion for prostate cancer and 2. Follow-up prostatectomy within 1 year of the prostate MRI. Exclusion criteria included 1. Patients who had simple prostatectomies performed for benign prostatic hyperplasia. 2. Hip or other orthopedic implants affecting the image quality of the prostate gland.

### Image Acquisition, Surgery, and Histopathologic Evaluation

All images were acquired on a variety of 3T MR systems with pelvic multiarray surface coil (Siemens MAGNETOM: Vida, Prisma, Skyra, Sola; Erlangen, Germany). All image acquisitions followed our standard clinical protocol which includes a small field-of-view axial T2 weighted image (T2WI), coronal T2WI, sagittal HASTE, DWI (b = 50 s/mm^2^, b = 1000 s/mm^2^), calculated high b value (b = 1500 s/mm^2^), apparent diffusion coefficient map (ADC), large field-of-view in and opposed phase T1 gradient, T1 without intravenous contrast, small field-of-view Golden-angle RAdial Sparse Parallel (GRASP), and large field-of-view post contrast T1 with fat suppression (Supplemental Table 1). The GRASP includes a single post contrast T1 fat suppressed sequence acquired over 3 minutes which is post-processed into a DCE with 3 sec time resolution [21]. Radical prostatectomies were performed by urology oncologists. Histopathology was reviewed by genitourinary pathologists, according to International Society of Urologic Pathology 2014 consensus statement [22]. The radical prostatectomy ISUP grade group and EPE including focal and nonfocal for each patient was recorded.

### Deep Learning Model for EPE

Prediction of EPE from prostate MRI is a data-limited problem, as reliable ground truth is primarily available from prostatectomy specimens and therefore only for a subset of patients. To address this limitation, we adopted a sequential training strategy in which a deep learning architecture was first trained on related prostate MRI tasks with substantially larger labeled datasets before being applied to EPE prediction (Fig. 1).

All models were implemented in PyTorch using a multi-branch 3D convolutional neural network based on a 3D ResNet-50 backbone. The network consisted of parallel input branches, each processing a distinct MRI input stream, followed by feature fusion and a shared classification head. Two versions of the model were trained and evaluated: one incorporating only T2WI and DWI (AI_bp_) and one additionally leveraging the single post contrast GRASP sequence (AI_mp_). In both versions, axial T2WI was processed through a dedicated branch. Diffusion information was provided through a second branch in which the ADC map and high b-value diffusion-weighted image (b = 1500 s/mm^2^) were stacked as separate input channels and jointly processed. In the DCE-enabled model (AI_mp_), an additional branch processed post-contrast DCE images. Feature maps from all branches were concatenated after the final convolutional stage.

Using this shared architecture, we trained a series of models on progressively more specific prostate cancer tasks. These intermediate tasks were chosen to encourage learning of prostate anatomy, lesion appearance, and cancer-related imaging features prior to training the EPE model. First, the image-based models were trained to predict the presence of PI-RADS ≥ 4 lesions at the case level using labels derived from structured radiology reports (38,000 cases). Second, the models were trained to predict clinically significant prostate cancer, defined as Gleason score ≥ 7, using histopathology as ground truth (7000 cases). Finally, the EPE models were trained to classify organ-confined disease (≤ T2) versus EPE (≥ T3) using surgical pathology with 1003, 113, 116 cases in the train, validation and test splits, respectively.

All models used identical imaging inputs and architecture, differing only in task-specific output layers and training labels. Models were trained using binary cross-entropy loss with the Adam optimizer, with early stopping based on AUC on the validation set. Data augmentation, including random flips and small affine perturbations, was applied during training.

### Logistic regression with PSA density

To assess complementarity with image-based predictions, we trained a logistic regression model incorporating the AI_mp_ model and prostate-specific antigen density (PSAD) (AI_mpPSAD_). PSA density was calculated as serum PSA divided by MRI-derived prostate volume. PSAD values were log-transformed prior to modeling. The logistic regression model was trained using maximum likelihood estimation with L2 regularization. Missing PSAD values in the training set (90/1003, 9%) were imputed using the median value from the training set. Model performance was evaluated on the same held-out test set as the deep learning models using AUC.

### Image Review

The same test set used to test the model, unique from the training and validation sets, was anonymized and randomized and presented to three abdominal fellowship trained radiologists (with > 5000, > 1800, and > 1700 prostate MRI experience). The readers evaluated the studies for the following characteristics of the dominant lesion: abutment of capsular length > 1.5 cm, capsular bulging, capsular irregularity, gross EPE. Based on these characteristics, the radiologist gave an EPE score of 1–5 as follows: 1 = no evidence of EPE, 2 = capsular abutment length > 1.5 cm, 3 = capsular bulging OR irregularity, 4 = capsular bulging AND irregularity, 5 = gross EPE. Prior to the study, the radiologists were shown examples of each. The readers first evaluated the non-contrast sequences. After noting the scores for the non-contrast sequences, the readers evaluated the entire study including the DCE and post-contrast sequences using the same grading scale.

### Statistics

Comparison between demographics of training set, validation set, and test set were performed with ANOVA test for continuous variables, and chi square test and Fisher exact test for ordinal variables. Area under the receiver operating characteristic curves (AUC) with 95% confidence intervals were obtained for the AI_bp_, AI_mp_, and AI_mpPSAD_. AUC was calculated for the reader scores evaluating bpMRI and mpMRI and for predictions of focal and nonfocal EPE. Comparisons were made between AUC curves using the Delong test. The Youden indices were also calculated for the receiver operating characteristic curves (ROC) of the deep learning models, and the diagnostic statistics were calculated including sensitivity, specificity, positive predictive value (PPV), negative predictive value (NPV), and accuracy. We matched the sensitivity of the AI_mp_ and AI_mpPSAD_ to that of AI_bp_ at the Youden index and calculated the associated diagnostic statistics for each. The same diagnostic statistics were calculated for reader results by dichotomizing the 5-point EPE scale with scores 1 and 2 marked as negative for EPE and 3 or greater marked as positive for EPE. Fleiss’ kappa was performed to assess inter-reader variability. The majority determination of the dichotomized EPE assignment between the three readers was used as a representation of all three readers when assessing discordances between models and readers. All statistical work was performed with R Studio (Posit PBC, Boston, MA; R Foundation, Vienna, Austria).

## Results

### Patients

A total of 1307 patients were initially included in the search. After exclusions, 1232 studies were included in the final study cohort with 653 (53%) with EPE (Fig. 2). 1003 (523 with EPE, 174 focal, 349 nonfocal) studies were used in the training set, 113 (57 with EPE, 21 focal, 36 nonfocal) were used in the validation set, and 116 (61 with EPE, 23 focal, 37 nonfocal) were used in the test set. The mean PSAD of all included patients was 0.23 ± 0.23 ng/mL^2^. The complete demographics of included patients are in Table 2.

### AI Performance

All AI and reader diagnostic statistics are presented in Table 3 and AUCs are presented in Table 4. The AUC of AI_bp_, AI_mp_, and AI_mpPSAD_ were 0.71 (confidence interval [CI] 0.62–0.81), 0.72 (CI: 0.62–0.82), and 0.73 (CI: 0.64–0.83) respectively (Fig. 3). The sensitivity, specificity, PPV, NPV, and accuracy at the Youden index for AI_bp_ was 0.77, 0.64, 0.70, 0.71, and 0.71, for AI_mp_ was 0.75, 0.69, 0.73, 0.72, and 0.72, and for AI_mpPSAD_ was 0.98, 0.25, 0.59, 0.93, and 0.64. When held at a constant sensitivity of 0.77, the specificity and accuracy increased between AI_bp_ (specificity 0.64, accuracy 0.71) and AI_mp_ (specificity 0.69, accuracy 0.73) and AI_mpPSAD_ (specificity 0.71, accuracy 0.74) (Table 3). The models had higher performance in detecting nonfocal EPE (AUC: AI_bp_ 0.75, AI_mp_ 0.78, AI_PSAD_ 0.80) versus focal EPE (AUC: AIbp 0.64, AImp 0.63, AIPSAD 0.63) (Fig. 4).

### Reader Performance

The readers’ AUC for evaluating bpMRI was as follows: Reader1: 0.69 (CI: 0.60–0.77), Reader2: 0.71 (CI: 0.62–0.80), and Reader3: 0.66 (CI: 0.56–0.75). The readers’ AUC for evaluating mpMRI was as follows: Reader1: 0.71 (CI: 0.62–0.80), Reader2: 0.74 (CI: 0.65–0.83), Reader3: 0.69 (CI: 0.60–0.78) (Table 3). The AUC of all readers also trended higher when evaluating mpMRI when compared to bpMRI. Compared to evaluating bpMRI, the readers all had increased sensitivity and accuracy and decreased specificity when evaluating mpMRI (Table 4). There was moderate agreement in the dichotomized detection of EPE on bpMRI (kappa = 0.50) and substantial agreement on mpMRI (kappa = 0.61).

### Comparison Between AI Models and Readers

Further investigation of the relationships between AI_bp_ versus AI_mp_, AI_bp_ versus Reader_bp_, and AI_mp_ versus Reader_mp_ revealed discordances. AI_mp_ had 12 “misses” (6 false positives (FPs) and 6 false negatives (FNs)) compared to AI_bp_. Of these FNs, 4 were of focal EPE and 2 were of nonfocal EPE. AI_bp_ had 10 “misses” (4 FPs and 6 FNs). Of the 6 FNs, 3 were focal EPE and 3 were nonfocal EPE. Reader_bp_ and Reader_mp_ were obtained by using the majority dichotomous rating of the presence of EPE. Compared to AI_bp_, Reader_bp_ had 18 “misses” (1 FPs and 17 FNs). Of the FNs, there were 12 focal and 5 nonfocal EPE. Compared to ReaderAI_bp_, AI_bp_ had 13 “misses” (10 FPs and 3 FNs). Of the FNs, there was 1 focal and 2 noncal EPE. Compared to AI_mp_, Reader_mp_ had 16 “misses” (4 FPs and 12 FNs). Of the FNs, there were 7 focal and 5 nonfocal EPE. Compared to Reader_mp_, AI_mp_ had 19 “misses” (12 FPs and 7 FNs). Of the FNs, there were 3 focal and 4 nonfocal EPE.

### Patient Examples

Supplemental Fig. 1 shows an example of a patient with correct positive EPE prediction from all AI models and readers. Figure 5 shows an example of a patient with correctly predicted EPE by the AI models but missed by all the readers.

## Discussion

This study investigated the performance of deep learning models on detecting EPE on bpMRI versus mpMRI in comparison to fellowship-trained readers. We found that the models performed similarly to readers. With the addition of contrast enhanced images, the readers showed improved sensitivity, accuracy, and inter-reader agreement. The addition of contrast enhanced images and PSAD slightly increased performance of AI models. Although there were small differences in performance across AI and readers, the differences were not significant.

Local staging is an important prognostic indicator and is essential for presurgical and pretreatment planning, thus, there has been great interest in creating an imaging-based automated system to identify EPE [23, 24]. Our model was unique in that it was a classification model with no input of prostate and tumor segmentation, only labels of Gleason grade group and presence of EPE both from prostatectomy pathology reports. Two recent systematic reviews have been performed for models to predict EPE included 21 studies in one and 8 in another [25, 26]. The included studies demonstrated a wide variety of approaches including machine learning with convolutional neural networks, radiomics, and multimodal models including clinical variables modeled after traditional nomograms [25, 26]. Of these, Hou et al, Moroianu et al, Priester et al, and Simon et al presented models which use convolutional neural networks and machine learning methodologies to analyze bpMRI through prostate gland and tumor labels as inputs [18, 19, 25-28].

Hou et al developed a model most similar to ours and used the largest dataset of 849 studies. The prostate gland and lesions were segmented and compared to histologic results from prostatectomies. Their model was 2D convolutional neural network (CNN), ResNeXt, with a convolutional block attention module (CBAM) which used the segmentations to focus the attention of the models, and the output of the model was likelihood of EPE. They initially trained with an input of a single slice with the largest area of the lesion. Their model had an output of a single slice and multi-slice prediction of EPE based on the segmentations. In contrast, we used a 3D CNN, ResNet, that took an input of the entire volume of the prostate without any segmentation of the prostate or lesions. This decreased the upfront time of data preparation. We used a multi-step transfer learning model to focus the algorithm first on PI-RADS 4 and 5 lesions, then on GG ≥ 2 labels, and finally on the presence of EPE. While our AI_bp performance (AUC = 0.71) was slightly lower than that reported in Hou et al (AUC 0.76), a direct comparison is not possible due to the models being evaluated on different sets [28].

Moroianu et al and Simon et al both used a combination of deep learning models built for prostate lesion identification and segmentation in combination with additional probabilistic or random forest models based on positioning of segmented lesions in conjunction with the prostate capsule [18, 19]. They achieved AUC of 0.70 and 0.71 respectively, similar to our results, however requiring much more data preparation. Priester et al used a multi-modal deep learning model with inputs of T2WI, PSA, biopsy locations, and biopsy pathology with an output of a tumor map [27]. EPE risk was estimated based on the probability of csPCa in the prostate gland capsule [27].

When compared to readers, our models demonstrated similar AUC with higher sensitivity and lower specificity. As shown in previous literature, readers often under call EPE, which is reflected in the larger number of false negatives in our study [29]. The largest differences in false negative numbers between the AI models and readers in our study were in the number of missed focal EPE, a lower volume EPE and expectedly less visible on imaging. In the clinical setting, the increased sensitivity in detecting EPE is a potential protection against undertreatment as the presence of EPE upgrades the staging of prostate cancer to high-risk or very high-risk group [23]. This is especially true in radiation treatment planning where the addition of androgen deprivation therapy is recommended with higher risk disease.

Our study also compared readers and models using bpMRI to mpMRI. Post contrast sequences have the potential to add value in the detection of EPE given the high contrast of enhancing tumor compared to the surrounding normal tissue [30, 31]. GRASP may be useful in addition to T2WI in detecting EPE by readers and deep learning models since it has high resolution and is motion robust [21, 31]. GRASP increased the specificity, PPV, NPV, and accuracy of the models when sensitivity was held constant. All readers had increased sensitivity and accuracy in detecting EPE when reading mpMRI compared to bpMRI. These consistent trends show that GRASP may add value, however, the sample size may have been too small to realize a statistical significance.

Clinical parameters are known to help with prostate cancer prognostication and prediction of EPE in traditional nomograms and in ones that incorporate prostate MRIs [15, 32]. We trained a logistic regression model incorporating PSAD to AI_mpMRI_. The results increased AUC slightly, but not significantly. It also slightly increased the specificity, PPV, NPV, and accuracy of AI_mpMRI_ when matching sensitivity. These small increases suggest that clinical parameters may help, but imaging features drive the model.

There were several limitations to this study. This is a single center retrospective study performed only on studies acquired from a single vendor, thus the generalizability was not assessed. We included post treatment patients who may have had prior androgen deprivation therapy, focal therapy, or radiation therapy. The numbers of patients who had prior treatment for prostate cancer was low and thus likely did not have significant impact on the model performance. Our ground truth labels for EPE were dichotomized to positive or negative. We recognize that evaluation of EPE is more nuanced histologically and that there are management differences to focal versus non-focal EPE. The readers assessed the mpMRI immediately after bpMRI for each study which could have biased the mpMRI reads. Given that DCE is less emphasized in PI-RADS, we felt that this would not deviate significantly from the way readers normally interpret studies. The model used only the single conglomerate GRASP and not a true reconstructed dynamic contrast series with multiple time points, while the readers had access to both. This may have given the readers a slight advantage as they were able to assess early enhancement, where the tumor may have had even more contrast against the normal prostate. Future directions include re-training these algorithms with additional data points such as the side of EPE, biopsy Gleason grade, and other pertinent clinical variables.

## Conclusion

We built a classification-based deep learning model that demonstrated no significant different EPE detection rates as abdominal fellowship trained radiologists. The addition of contrast enhanced sequences slightly increased AUC for all the readers more than for the model, though the differences were not significant.

## Supplementary Material

This is a list of supplementary files associated with this preprint. Click to download.

• SupplementalMaterials.docx

## Figures and Tables

**Figure 1:**
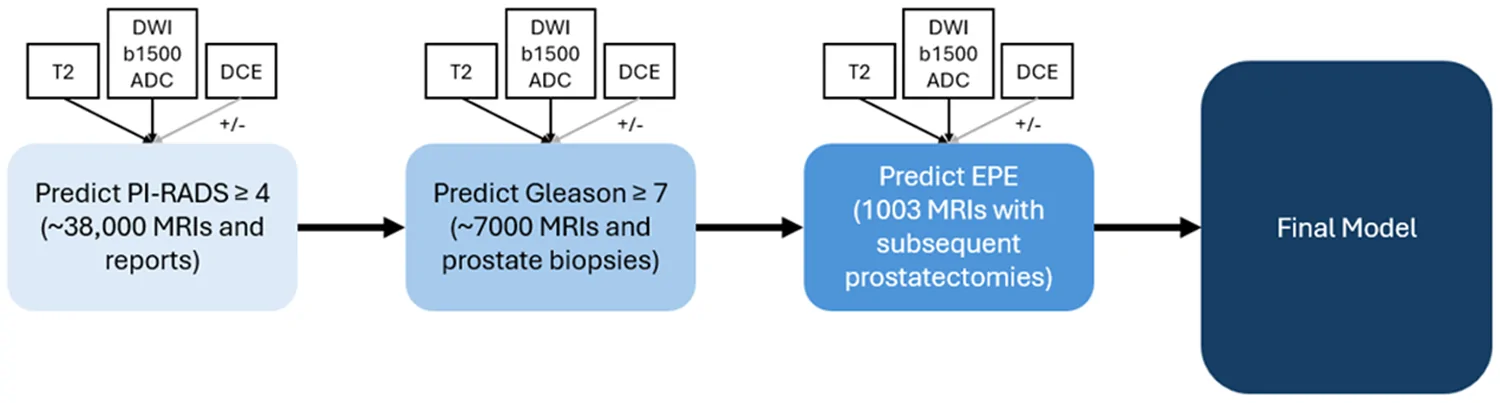
Transfer learning was used to build our image-based models. The models are first trained to predict Prostate Imaging Reporting and Data System (PI-RADS) ≥ 4, then trained to predict Gleason ≥ 7, and finally to predict extraprostatic extension (EPE). Inputs in each section include T2, diffusion weighted imaging (DWI) including high b-value sequence (b1500 s/mm^2^) and apparent diffusion coefficient (ADC), and with and without dynamic contrast enhanced imaging (DCE).

**Figure 2:**
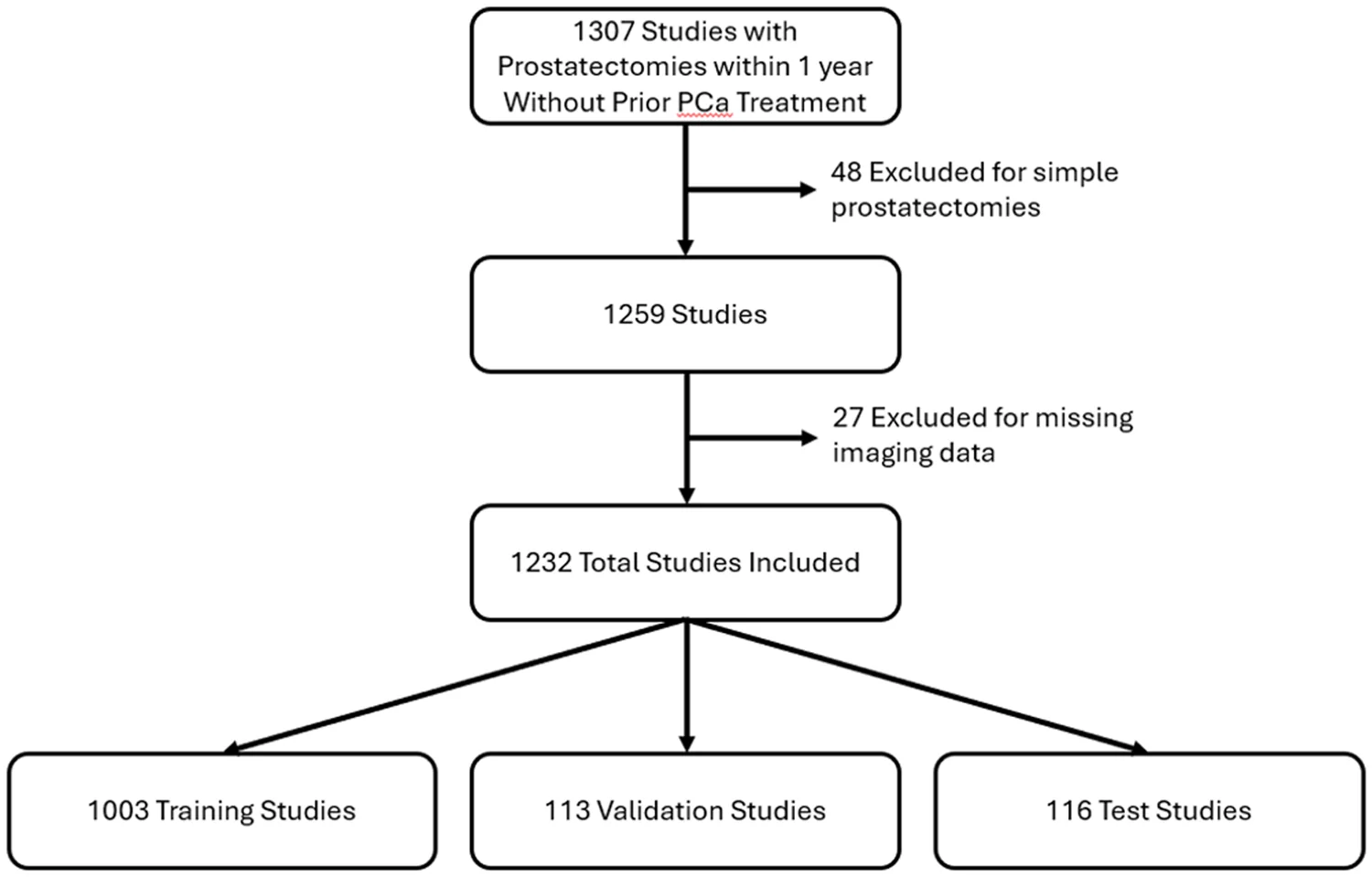
Flow chart of Patient Cohort.

**Figure 3:**
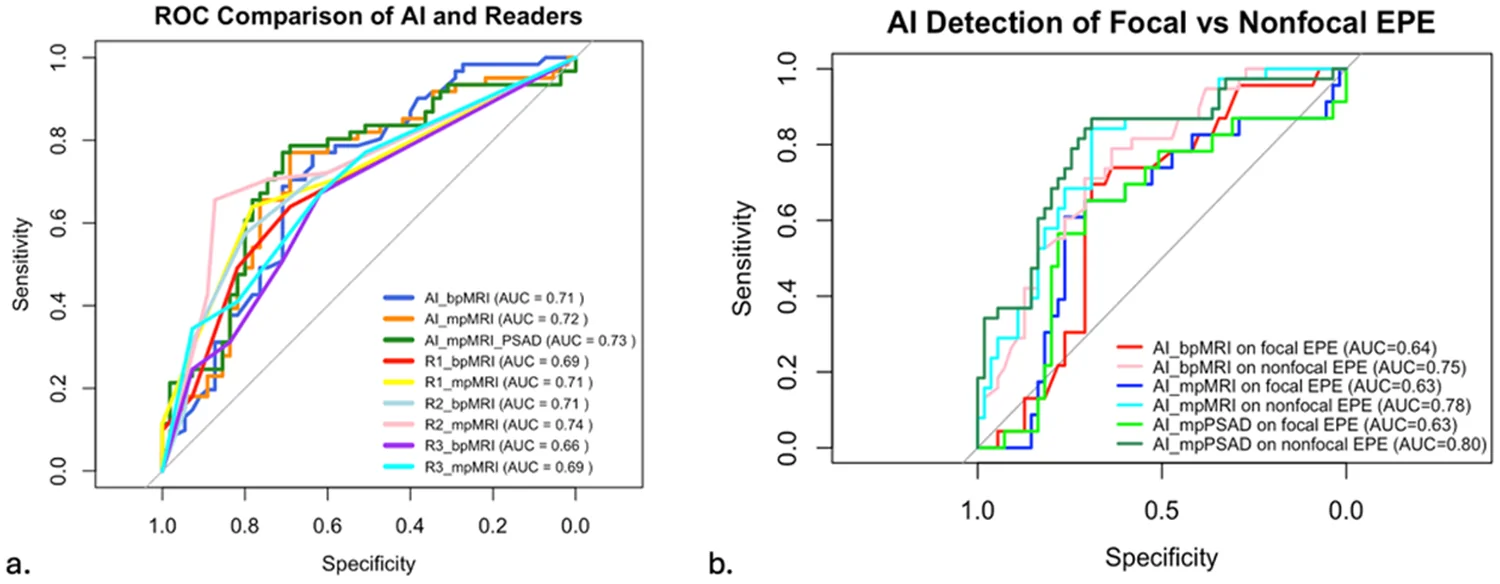
a. Receiver operating characteristic curves of the deep learning models using biparametric MRI (bpMRI), multiparametric MRI (mpMRI), and mpMRI with PSAD, and readers evaluating bpMRI and bpMRI. All demonstrate similar area under the curve without significant differences. b. Receiver operating characteristic curves of deep learning models comparing focal and nonfocal EPE and

**Figure 4:**
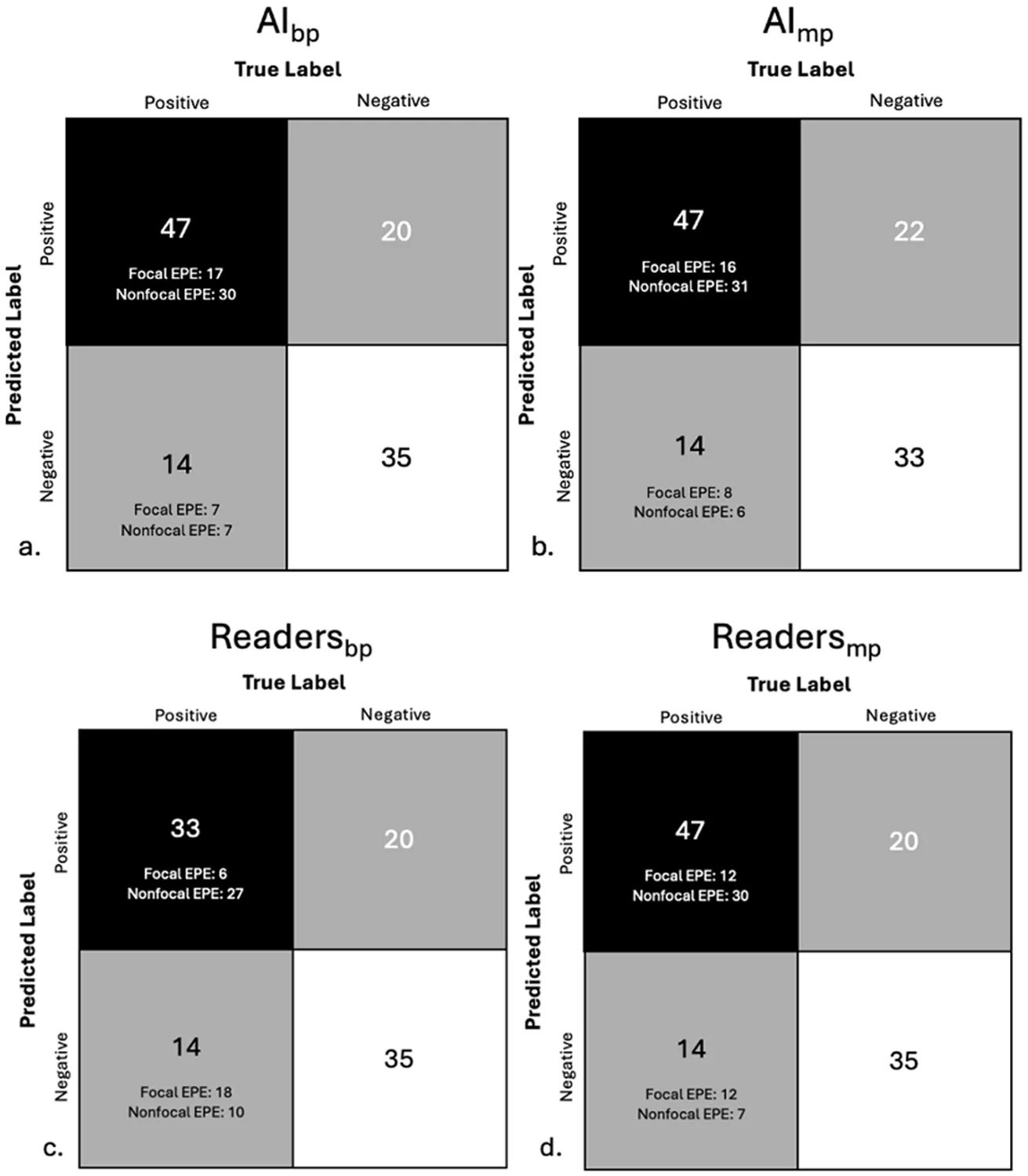
Confusion matrix of the AI algorithms and readers in comparison to histology results from prostate biopsies in the test set including a) AI_bp_, b) AI_mp_, c) Readers_bp_, and d) Readers_mp_.

**Figure 5:**
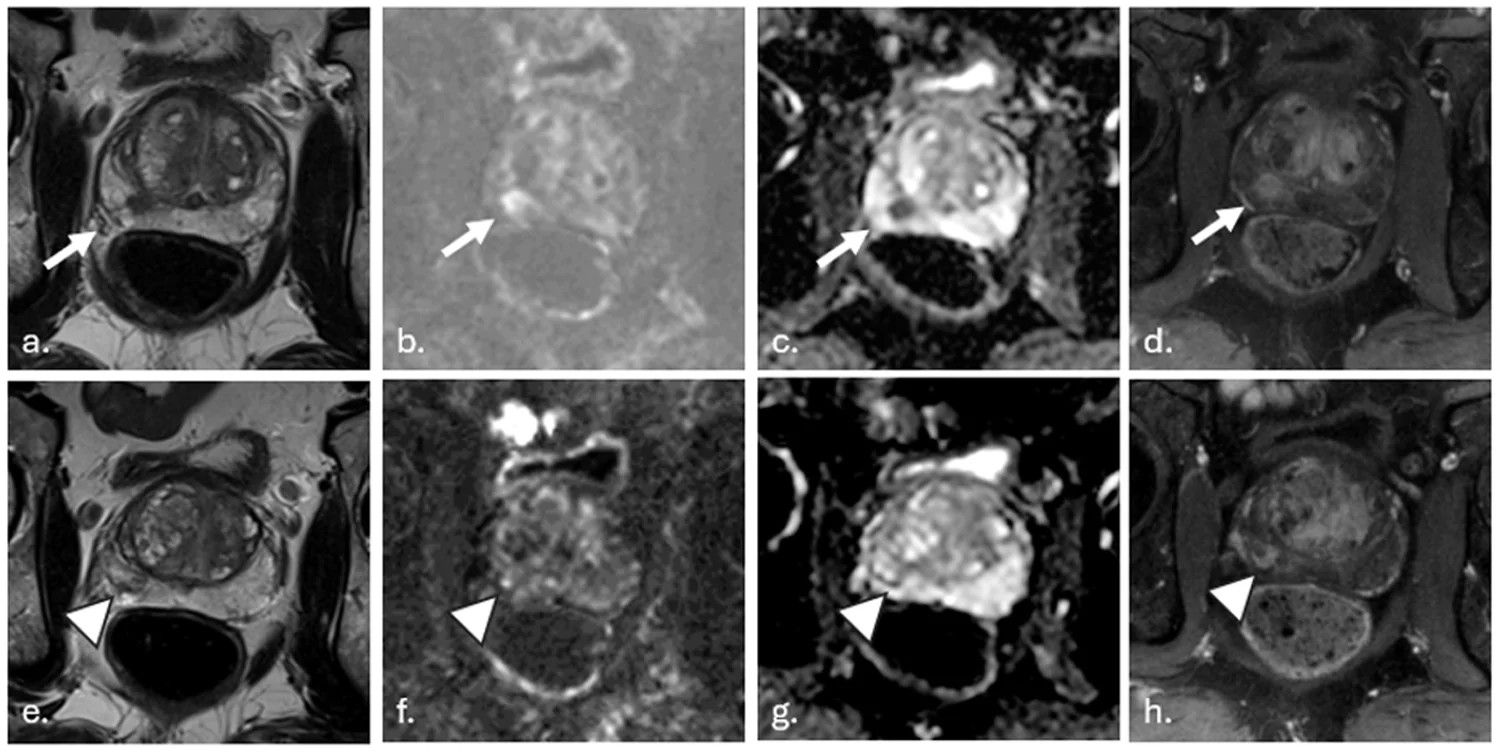
A 66 year-old patient presented with Gleason grade group 2 prostate cancer with PSA 9.12 ng/mL and had a PI-RADS 4 lesion in the right posterolateral midgland peripheral zone (arrow). None of the readers detected extraprostatic extension (EPE) (arrow head), however, each of the AI models identified EPE on this lesion when using the Youden index cutoff.

**Table 1:** Prostate MRI Scanning Parameters

Sequence	Slice thickness (mm)	TR (ms)	TE (ms)	Flip Angle	In-plane resolution (mm x mm)
**Ax T2 TSE**	3	4000	100	160	0.6 x 0.8
**Cor T2 TSE**	3	400	100	160	0.6 x 0.8
**Sag HASTE**	3	1400	150	120	0.7 x 1.0
Ax DWI (b50, 1000)*	3	5600	77	90	0.5 x 0.5
**Ax In/Opposed Phase**	5	120	1.2/2.4	52	1.5 x 1.5
**Ax Vibe (pre and post contrast)**	3	2.7	1.0	12	1.5 x 2.2
**Ax GRASP DCE**	3	4.1	2.0	12	0.8 x 0.8

TR = repetition time, TE = echo time, Ax = axial, TSE = turbo spin echo, Cor = coronal, Sag = sagittal, HASTE = Half-Fourier Acquisition Single shot Turbo spin-Echo, DWI = diffusion weighted imaging, GRASP = golden angle radial sparse parallel, DCE = dynamic contrast enhanced imaging

**Table 2:** Demographics and Dataset Descriptions

Category	Training Set (n = 1003)	Validation Set (n = 113)	Test set (n = 116)	P
**Mean Age (Std Dev)**	63.8 (7.2)	63.5 (7.0)	63.7 (7.1)	0.91
**Prostate Volume (Std Dev)**	43.6 (26.8)	41.5 (19.5)	43.1 (22.4)	0.72
**PSA (Std Dev)**	8.5 (8.7)	8.4 (9.1)	7.8 (6.5)	0.73
**PSA Density (Std Dev)**	0.24 (0.34)	0.20 (0.25)	0.23 (0.15)	0.48
**Gleason Grade Group 1 (% total)** *	33 (3.3)	3 (2.7)	5 (4.3)	0.82
**Gleason Grade Group 2 (% total)** *	516 (51.4)	59 (52.2)	63 (54.3)	0.95
**Gleason Grade Group 3 (% total)** *	275 (27.4)	36 (31.9)	33 (28.4)	0.76
**Gleason Grade Group 4 (% total)** *	49 (4.9)	2 (1.8)	1 (0.9)	0.06
**Gleason Grade Group 5 (% total)** *	124 (12.4)	11 (9.7)	12 (10.3)	0.76
**Post Radiation Treatment (% total)**	2 (0.2)	0	0	1.00
**Post ADT (% total)**	4 (0.4)	2 (1.8)	2 (1.7)	0.05
**EPE (% total)**	534 (53.2)	58 (51.3)	61 (52.6)	0.98
**Focal EPE (% total)**	174 (17.3)	21 (18.6)	23 (19.8)	0.78
**Nonfocal EPE (% total)**	349 (34.8)	36 (31.9)	38 (32.8)	0.77

Std Dev = standard deviation, PSA = prostate specific antigen, ADT = androgen deprivation therapy, EPE = extraprostatic extension

* Gleason Grade Group is based on prostatectomy results

**Table 3:** Diagnostic Statistics of Detecting Extraprostatic Extension of Deep Learning Models and Readers

AI bpMRI *	Sensitivity	Specificity	PPV	NPV	Accuracy
	0.77	0.64	0.70	0.71	0.71
AI mpMRI*	0.77	0.60	0.68	0.70	0.69
AI mp + PSAD*	0.77	0.71	0.75	0.74	0.74
R1 bpMRI	0.49	0.82	0.75	0.59	0.64
R1 mpMRI	0.66	0.78	0.77	0.67	0.72
R2 bpMRI	0.57	0.80	0.76	0.63	0.68
R2 mpMRI	0.70	0.75	0.75	0.69	0.72
R3 bpMRI	0.51	0.71	0.66	0.57	0.60
R3 mpMRI	0.67	0.62	0.66	0.63	0.65
Maj Reader bpMRI	0.54	0.80	0.75	0.61	0.68
Maj Reader mpMRI	0.69	0.75	0.75	0.68	0.72

* Matched to sensitivity of AI bpMRI at the Youden index. bpMRI = biparametric MRI, mpMRI = multiparametric MRI, PSAD = prostate specific antigen density, R1 = reader 1, R2 = reader 2, R3 = reader 3, PPV = positive predictive value, NPV = negative predictive value, Maj Reader bpMRI = conglomerate reader results based on majority binary extraprostatic extension interpretation on bpMRI, Maj Reader mpMRI = conglomerate reader results based on majority binary extraprostatic extension interpretation on mpMRI

**Table 4:** Comparison of the Area Under the Receiver Operating Characteristic Curve Between Models and Radiologists

Model/Rad	AUC (CI)	Model/Rad	AUC (CI)	p
AI_bp_	0.71 (0.62–0.81)	AI_mp_	0.72 (0.62–0.81)	0.82
AI_bp_	0.71 (0.62–0.81)	AI_mpPSAD_	0.73 (0.64–0.83)	0.52
AI_mp_	0.72 (0.62–0.81)	AI_mpPSAD_	0.73 (0.64–0.83)	0.09
AI_bp_	0.71 (0.62–0.81)	R1_bp_	0.69 (0.60–0.77)	0.56
AI_bp_	0.71 (0.62–0.81)	R1_mp_	0.71 (0.62–0.80)	0.97
AI_bp_	0.71 (0.62–0.81)	R2_bp_	0.71 (0.62–0.80)	1.00
AI_bp_	0.71 (0.62–0.81)	R2_mp_	0.74 (0.65–0.83)	0.53
AI_bp_	0.71 (0.62–0.81)	R3_bp_	0.66 (0.56–0.75)	0.27
AI_bp_	0.71 (0.62–0.81)	R3_mp_	0.69 (0.60–0.78)	0.68
R1_bp_	0.69 (0.60–0.77)	R1_mp_	0.71 (0.62–0.80)	0.32
R2_bp_	0.71 (0.62–0.80)	R2_mp_	0.74 (0.65–0.83)	0.11
R3_bp_	0.66 (0.56–0.75)	R3_mp_	0.69 (0.60–0.78)	0.17

AI_bp_ = deep learning model trained on biparametric MRI (bpMRI), AI_mpMRI = deep learning model trained on multiparametric MRI (mpMRI), AI_mp_PSAD = deep learning model trained on mpMRI and prostate specific antigen density, R1 = reader 1, R2 = reader 2, R3 = reader 3

## Data Availability

The data that support the findings of this study are not openly available due to reasons of sensitivity and are available from the corresponding author upon reasonable request. Data are located in controlled access data storage at NYU Langone Health.

## References

[1] Kratzer T.B., , Prostate cancer statistics, 2025. CA Cancer J Clin, 2025. 75(6): p. 485–497. 10.3322/caac.70028 PMC12593258 40892160

[2] Lee C.H., Akin-Olugbade O., and Kirschenbaum A., Overview of prostate anatomy, histology, and pathology. Endocrinol Metab Clin North Am, 2011. 40(3): p. 565–75, viii-ix. 10.1016/j.ecl.2011.05.012 21889721

[3] DeMarzo A.M., , Pathological and molecular aspects of prostate cancer. The Lancet, 2003. 361(9361): p. 955–964. 10.1016/S0140-6736(03)12779-1 12648986

[4] Shieh A.C., , Extraprostatic extension in prostate cancer: primer for radiologists. Abdominal Radiology, 2020. 45(12): p. 4040–4051. 10.1007/s00261-020-02555-x 32390076

[5] Schaeffer E.M., , Prostate Cancer, Version 4.2023, NCCN Clinical Practice Guidelines in Oncology. Journal of the National Comprehensive Cancer Network, 2023. 21(10): p. 1067–1096. 10.6004/jnccn.2023.0050 37856213

[6] Pisansky T.M., , Adjuvant and Salvage Radiotherapy after Prostatectomy: ASTRO/AUA Guideline Amendment 2018–2019. Journal of Urology, 2019. 202(3): p. 533–538. 10.1097/JU.0000000000000295 PMC8680266 31042111

[7] Sekito S., , Predictive Factors for Extracapsular Extension of Prostate Cancer to Select the Candidates for Nerve-sparing Radical Prostatectomy. Indian J Surg Oncol, 2024. 15(2): p. 213–217. 10.1007/s13193-024-01913-1 PMC11088566 38741620

[8] Bill-Axelson A., , Radical Prostatectomy or Watchful Waiting in Prostate Cancer — 29-Year Follow-up. New England Journal of Medicine, 2018. 379(24): p. 2319–2329. 10.1056/NEJMoa1807801 30575473

[9] Kasivisvanathan V., , MRI-Targeted or Standard Biopsy for Prostate-Cancer Diagnosis. N Engl J Med, 2018. 378(19): p. 1767–1777. 10.1056/NEJMoa1801993 PMC9084630 29552975

[10] Rouviere O., , Use of prostate systematic and targeted biopsy on the basis of multiparametric MRI in biopsy-naive patients (MRI-FIRST): a prospective, multicentre, paired diagnostic study. Lancet Oncol, 2019. 20(1): p. 100–109. 10.1016/S1470-2045(18)30569-2 30470502

[11] Zhang F., , Accuracy of multiparametric magnetic resonance imaging for detecting extracapsular extension in prostate cancer: a systematic review and meta-analysis. Br J Radiol, 2019. 92(1104): p. 20190480. 10.1259/bjr.20190480 PMC6913368 31596123

[12] Triquell M., , Multiparametric MRI for Staging of Prostate Cancer: A Multicentric Analysis of Predictive Factors to Improve Identification of Extracapsular Extension before Radical Prostatectomy. Cancers, 2022. 14(16): p. 3966. 10.3390/cancers14163966 PMC9406654 36010963

[13] Somford D.M., , The Predictive Value of Endorectal 3 Tesla Multiparametric Magnetic Resonance Imaging for Extraprostatic Extension in Patients with Low, Intermediate and High Risk Prostate Cancer. Journal of Urology, 2013. 190(5): p. 1728–1734. 10.1016/j.juro.2013.05.021 23680307

[14] Ng A.B.C.D., , Biparametric vs Multiparametric MRI for Prostate Cancer Diagnosis. JAMA, 2025. 334(13): p. 1170. 10.1001/jama.2025.13722 PMC12423955 40928788

[15] Mehralivand S., , A Grading System for the Assessment of Risk of Extraprostatic Extension of Prostate Cancer at Multiparametric MRI. Radiology, 2019. 290(3): p. 709–719. 10.1148/radiol.2018181278 PMC6394788 30667329

[16] Gatti M., , mEPE-score: a comprehensive grading system for predicting pathologic extraprostatic extension of prostate cancer at multiparametric magnetic resonance imaging. European Radiology, 2022. 32(7): p. 4942–4953. 10.1007/s00330-022-08595-9 PMC9213375 35290508

[17] Cuocolo R., , MRI index lesion radiomics and machine learning for detection of extraprostatic extension of disease: a multicenter study. European Radiology, 2021. 31(10): p. 7575–7583. 10.1007/s00330-021-07856-3 PMC8452573 33792737

[18] Moroianu Ş.L., , Computational Detection of Extraprostatic Extension of Prostate Cancer on Multiparametric MRI Using Deep Learning. Cancers, 2022. 14(12): p. 2821. 10.3390/cancers14122821 PMC9220816 35740487

[19] Simon B.D., , Automated Detection and Grading of Extraprostatic Extension of Prostate Cancer at MRI via Cascaded Deep Learning and Random Forest Classification. Academic Radiology, 2024. 31(10): p. 4096–4106. 10.1016/j.acra.2024.04.011 PMC11490411 38670874

[20] Jeganathan T., , Inter-Rater Variability of Prostate Lesion Segmentation on Multiparametric Prostate MRI. Biomedicines, 2023. 11(12). 10.3390/biomedicines11123309 PMC10741937 38137530

[21] Rosenkrantz A.B., , Dynamic contrast-enhanced MRI of the prostate with high spatiotemporal resolution using compressed sensing, parallel imaging, and continuous golden-angle radial sampling: preliminary experience. J Magn Reson Imaging, 2015. 41(5): p. 1365–73. 10.1002/jmri.24661 PMC4233205 24833417

[22] Epstein J.I., , The 2014 International Society of Urological Pathology (ISUP) Consensus Conference on Gleason Grading of Prostatic Carcinoma Definition of Grading Patterns and Proposal for a New Grading System. AMERICAN JOURNAL OF SURGICAL PATHOLOGY, 2016. 40(2): p. 244–252. 10.1097/PAS.0000000000000530 26492179

[23] Moses K.A., , NCCN Guidelines^®^ Insights: Prostate Cancer Early Detection, Version 1.2023. Journal of the National Comprehensive Cancer Network, 2023. 21(3): p. 236–246. 10.6004/jnccn.2023.0014 36898362

[24] Kumar A., , Prognostic significance of magnetic resonance imaging-detected extraprostatic extension in localized prostate cancer. Cancer, 2025. 131(23): p. e70191. 10.1002/cncr.70191 41288326

[25] Zhang X., , Artificial Intelligence-Enabled Imaging for Predicting Preoperative Extraprostatic Extension in Prostate Cancer: Systematic Review and Meta-Analysis. J Med Internet Res, 2025. 27: p. e80981. 10.2196/80981 PMC12688051 41364797

[26] Wu X. and Yao W., Artificial Intelligence Models Integrating Preoperative Prostate MRI and Clinical Parameters for Predicting Extraprostatic Extension: A Systematic Review and Meta-Analysis. J Surg Oncol, 2026. 10.1002/jso.70167 41588994

[27] Priester A., , Extracapsular extension risk assessment using an artificial intelligence prostate cancer mapping algorithm. BJUI Compass, 2024. 5(10): p. 986–997. 10.1002/bco2.421 PMC11479810 39416757

[28] Hou Y., , Artificial intelligence is a promising prospect for the detection of prostate cancer extracapsular extension with mpMRI: a two-center comparative study. Eur J Nucl Med Mol Imaging, 2021. 48(12): p. 3805–3816. 10.1007/s00259-021-05381-5 34018011

[29] Park K.J., Kim M.H., and Kim J.K., Extraprostatic Tumor Extension: Comparison of Preoperative Multiparametric MRI Criteria and Histopathologic Correlation after Radical Prostatectomy. Radiology, 2020. 296(1): p. 87–95. 10.1148/radiol.2020192133 32368959

[30] Winkel D.J., , High spatiotemporal resolution dynamic contrast-enhanced MRI improves the image-based discrimination of histopathology risk groups of peripheral zone prostate cancer: a supervised machine learning approach. Eur Radiol, 2020. 30(9): p. 4828–4837. 10.1007/s00330-020-06849-y 32328763

[31] Winkel D.J., , Compressed Sensing Radial Sampling MRI of Prostate Perfusion: Utility for Detection of Prostate Cancer. Radiology, 2019. 290(3): p. 702–708. 10.1148/radiol.2018180556 PMC6924615 30599102

[32] Wibmer A.G., , International Multi-Site Initiative to Develop an MRI-Inclusive Nomogram for Side-Specific Prediction of Extraprostatic Extension of Prostate Cancer. Cancers (Basel), 2021. 13(11). 10.3390/cancers13112627 PMC8198352 34071842

